# Affordability of commonly prescribed antibiotics in a large tertiary teaching hospital in Ethiopia: a challenge for the national drug policy objective

**DOI:** 10.1186/s13104-018-4021-2

**Published:** 2018-12-27

**Authors:** Girma Gutema, Ephrem Engidawork

**Affiliations:** 1 East African Policy Research Institute, Addis Ababa, Ethiopia; 2 University of Oslo, Oslo, Norway; 3 Department of Pharmacology and Clinical Pharmacy, School of Pharmacy, Addis Ababa University, Addis Ababa, Ethiopia

**Keywords:** Affordability, Policy, Antibiotics, TASH, Ethiopia

## Abstract

**Objective:**

In national drug policies of many countries, ensuring availability and affordability of essential medicines is indicated among the major policy objectives. To achieve the objectives, countries with low and middle income compile such medicines into NEMLs. This study aims to determine availability and affordability of commonly prescribed antibiotics at a tertiary hospital in Ethiopia by assessing (in private and public pharmacies) 13 antibiotics constituting DU90% at the hospital.

**Results:**

Availability of the antibiotics in the private and public pharmacies was 92.3% and 98.5%, respectively. Average MPRs for the antibiotics were 4.1 and 2.7, respectively, in the private and public pharmacies. The days’ wages (in median prices) ranged from 0.2 for treating acute diarrhea with doxycycline to 415.8 for treating HAP in public pharmacies. Costs of a single day treatment with antibiotics purchased from the public pharmacies ranged from USD 0.1 for acute diarrhea to USD 29.7 for HAP. For the private pharmacies, the range was from USD 0.1 for toxoplasmosis to USD 54.9 for HAP. This study showed that treatments of commonly diagnosed infectious conditions at TASH remain unaffordable according to the WHO/HAI criteria.

**Electronic supplementary material:**

The online version of this article (10.1186/s13104-018-4021-2) contains supplementary material, which is available to authorized users.

## Introduction

In the national drug policy of countries, ensuring availability and affordability of essential medicines are often placed as the main objectives. To achieve this, many low/middle-income countries have National Essential Medicines Lists (NEMLs) [1]. According to WHO, over 156 countries have NEMLs [2]. WHO recommends at least 80% availability of essential medicines in healthcare facilities [3]. However, in low/middle income countries this is still a major challenge [4]. In a study conducted in 36 countries, average availability of essential medicines was reported to be 38% and 64% in the public and private sector, respectively [5]. In Sudan, availability of medicines that matches with WHO’s recommendations has been reported [6]. In Malawi, 31% mean availability was reported in the public sector for antibiotics [7]. Sub-optimal availability of other essential medicines has also been reported elsewhere [8–11].

Ethiopian national drug policy aims to ensure provision of medicines with prices compatible with peoples’ purchasing power [12]. According to WHO’s ideal drug prescribing indicator, 100% of drugs prescribed should be from the NEML [13]. To enhance availability and affordability of those essential medicines in Ethiopia’s NEML [14], a government-supported Revolving Drug Fund (RDF) was established [15]. RDF involves a system of financing supply of medicines in which after an initial capital investment, drug supplies are replenished with the money collected from the sales of drugs [16]. However, studies showed results discordant with the RDF’s scheme [17–19].

Essential medicines are generally less affordable in the private than in public sector [17, 20]. Besides improving total health of population, enhanced affordability of essential medicines enhances productivity thereby playing positive roles in reducing poverty [21, 22].

Ethiopia has no functioning health insurance system. The recently introduced community based health insurance system being on a pilot stage [23], the vast majority of patients have to still pay for their medication and other healthcare costs. There is however a waiver system for what the government calls “the poorest of the poor”, although availability is still an issue [18]. According to the World Bank, 38.5% of the Ethiopian healthcare expenditures are covered by patients’ out of pocket payments [24]. Furthermore, about 33% of the population in the country live below the international poverty line of USD 1.9/day [25].

Antibiotics are among the most commonly prescribed drugs in Ethiopian hospitals as in most developing countries [26–28]. Two recent studies showed high prevalence of antibiotic use [28, 29]. Our previous study showed that overall, 14 antibiotic agents constituted the Drug Utilization 90% (DU90%) in three wards, of which 13 were listed in Ethiopia’s NEML [28].

Studies conducted on the availability and affordability of essential medicines can help identify gaps for policy interventions. In the present study, we assessed availability and affordability of the 13 commonly prescribed antibiotics from Tikur Anbessa Specialized Hospital (TASH).

## Main text

### Methods

#### Selection of pharmacies

The pharmacies were selected by adopting the WHO/HAI methodology [30]. By selecting TASH as reference, 14 pharmacies (5 from public and 9 from private sectors) in the area were visited for data collection in April 2015. In all pharmacies, the chief pharmacist provided the information on the availability and prices of the surveyed medicines. Data collection was done over five working days (April 13 to 17, 2015). The first 2 days, data for three public and six private pharmacies were collected. The last 3 day, data for two public and three private pharmacies were collected.

#### Data entry and analysis

Data entered into WHO/HAI’s MS Excel workbook. Availability was measured as percentage of facilities which had unexpired stock of the respective drugs in the commonly prescribed strength and dosage on the day of data collection. The Ethiopian Standard Treatment Guidelines for General Hospitals, 3rd edition [31] and the guidelines of the Infectious Disease Society of America (IDSA) [32] were benchmarked to assess treatment affordability. Accordingly, costs of treatment using the selected antibiotics when used as the first line or alternative treatment option were determined. The International Reference Prices (IRP) of 2014, provided by Management Science for Health (MSH) [33], were used for comparison with local prices of the antibiotics. For the purpose of comparison, prices were expressed as median price ratio (MPR), which is the ratio of median local unit price relative to IRP. To show price variations for each drug product, ‘high to low’ price ratios were determined. Affordability was measured according to the WHO/HAI methodology which states that a drug is considered unaffordable if more than 1 day’s wage is spent to pay for it, when used to treat an acute disease, by the lowest paid unskilled government employee. The costs of antibiotics for a full course of therapy according to the guidelines were calculated and changed to the day’s wages. During the study period, the minimum daily wage of an unskilled Ethiopian government employee was 20.5 Ethiopian Birr (ETB) per day. Since 20.8 ETB = 1USD, which was the average exchange rate for the month April 2015 at the Commercial Bank of Ethiopia, this was used for all calculations [34]. In addition, the medication cost for a single day treatment for the commonly encountered infections were determined and compared with World Bank’s under poverty-line for Ethiopia (USD 1.9).

### Results

Availability of the 13 commonly prescribed antibiotics in TASH was on average 92.3% and 98.5% in the private and public pharmacies, respectively. The range for the individual products was 77.8–100% in the private pharmacies, while 12 out of 13 products were available in all the public pharmacies (Additional file 1: Table S1 for more).

Average MPRs were 4.1 and 2.7, respectively, in the private and public pharmacies. In the public pharmacies, MPR ranged from 0.6 for ceftriaxone to 8 for metronidazole. In the private, the range was from 1 for erythromycin to 10 for doxycycline (Table 1).

**Table 1:** Median local price/unit, international reference price (USD) and median price ratios (MPR) of antibiotics in selected private and public pharmacies

Antibiotics	Unit	Median price per unit (USD)		International reference price (USD)	Median price ratio (MPR)	
		Private (n = 9)	Public (n = 5)		Private	Public
Ceftriaxone	1000 mg Vial	1.2	0.5	0.8	1.5	0.6
Metronidazole	500 mg Vial	0.9	0.8	0.1	9	8
Ciprofloxacin	500 mg Tab	0.1	0.1	0.04	2.5	2.5
Vancomycin	500 mg Vial	6.3	3.9	1.8	3.5	2.2
Co-trimoxazole	80/400 mg Tab	0.02	0.02	0.01	2	2
Azithromycin	500 mg Tab	1.2	1.3	0.3	4	4.3
Ampicillin	1000 mg Vial	0.9	0.3	0.2	4.5	1.5
Ceftazidime	1000 mg Vial	9.9	4.7	1.1	9	4.3
Gentamycin	80 mg/2 ml Amp	0.2	0.2	0.1	2	2
Amoxicillin	500 mg Cap	0.1	0.1	0.03	3.3	3.3
Cloxacillin	500 mg Vial	0.3	0.2	0.2	1.5	1
Doxycycline	100 mg Cap	0.1	0.03	0.01	10	3
Erythromycin	500 mg Tab	0.1	0.1	0.1	1	1

**Fig. 1:**
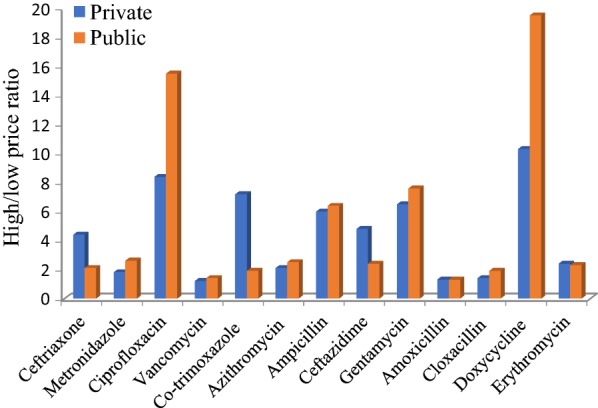
High to low price ratios of 13 commonly prescribed antibiotics in selected pharmacies

Figure 1 shows the high to low price ratios of the antibiotics in the public and private pharmacies.

Days’ wages ranged from 0.2 for treating acute diarrhea with doxycycline to 415.8 for treating HAP in public pharmacies. In private, the range was from 0.6 to 768.6 for treating same infections (Additional file 2: Table S2 for more).

Costs of single day treatment with antibiotics purchased from public pharmacies ranged from USD 0.1 for acute diarrhea to USD 29.7 for HAP. For those in private, the range was from USD 0.1 for toxoplasmosis to 54.9 for HAP (Table 2).

**Table 2:** Cost of single day treatment (USD) for commonly diagnosed infections at TASH with antibiotics purchased from public and private pharmacies (median prices)

Infection	Daily cost of antibiotics for standard treatment (in USD)	
	Public pharmacies	Private pharmacies
Sub-acute bacterial endocarditis (SBE)	1.2	2.6
Amoebic liver abscess	3.2	3.6
UTI-acute, uncomplicated	0.2	0.2
Meningitis	17.5	30
Sepsis	0.8	1.2
Community Acquired Pneumonia (CAP)	1.0	2.4
Hospital Acquired Pneumonia (HAP)	29.7	54.9
Spontaneous bacterial peritonitis	1.0	2.4
Diarrhea (when antibiotic treatment indicated)	0.1	0.2
Rheumatic fever	0.8	0.7
Toxoplasmosis	0.1	0.1

### Discussion

This study showed that availability is not a problem. Unexpected was however the higher availability of antibiotics in public pharmacies as compared with the private ones. Yet, a concern with availability might be the problem of counterfeited drug markets in developing countries [35]. Given infectious diseases account for major public health burden, availability of counterfeited/sub-standard quality antibiotics in the market would be anticipated [36]. Recently, some initiatives involving international collaborations aimed at tackling such problems have been started [37, 38].

Most studies on availability of essential drugs are national, regional/provincial surveys [39, 40]. Hence, it might be difficult to compare their findings with the present study. However, compared to a study done elsewhere in Ethiopia with limited geographic area like the present one [19], availability in the present study is higher.

Concerning affordability, it is not unexpected that antibiotics were found to be more expensive in private pharmacies, as also reported elsewhere [17, 41]. Price variations along sector facilities are high—as high as over 10 and 19 folds in the private and public pharmacies, respectively, for doxycycline, for instance. Drug prices are not controlled/regulated in Ethiopia and there is no agreed upon method or legally enforceable mechanism to determine the final/patient level price of drugs. But most public pharmacies have old tradition of tagging their prices by adding 25% profit margin on each product. Therefore, products imported from various countries can have wide price variations especially in private pharmacies [42]. With few exceptions, antibiotics were being sold at prices higher than IRPs. Price variations for the antibiotic products are larger in the private pharmacies and this could be because of lack of enforced price markups, control mechanisms and also market competition [17].

It is generally difficult to determine the cut-off point MPRs, as measure of affordability, because of various factors. However, WHO’s expert group [43] suggested MPR ≤ 1.5 and MPR ≤ 2.5 as acceptable cut-off points for the public and private sectors, respectively. Hence, if seen in light of these, this study showed that local prices of the following antibiotics are unacceptable: doxycycline, amoxicillin, ceftazidime, azithromycin, vancomycin, metronidazole (in both sectors), ciprofloxacin, co-trimoxazole, gentamycin (in public sector), ampicillin (in private sector). A 2012 UN report indicated that average MPRs were 5.3 and 3.1 in the private and public sectors, respectively, for low/middle income countries [44]. In this study, average MPRs for antibiotics included in the analysis were 4.1 and 2.7, respectively, in the private and public pharmacies.

In this study, treatment affordability for the common infections varied depending on whether the antibiotics were to be purchased from the private or public pharmacies. Taking into account WHO/HAI’s recommendation on affordability of essential medicines, most of the treatments were found to be unaffordable for the lowest paid unskilled government employee. Overall, among the 11 commonly diagnosed infections at TASH from the data in our earlier study [28], (full analysis results in Additional file 2: Table S2), standard treatments for 9 of them (81.8%) were unaffordable both in the private and public sectors.

Treatment affordability could further be a challenge because of two important factors. First, other costs involved in the treatment like costs of consultation and diagnostic tests are not included. When added to medicines’ costs, these put great economic burden on patients. Second, there are chances where more than one member within a family might get ill at the same time. Especially with infections, Ethiopia’s average family size in a household being 4.6 persons [45], there is real possibility that more than one members within a family could get ill at the same time, which makes it extremely difficult to afford the medications.

Benchmarking costs of treatments for common infections in the current study with World Bank’s data on poverty headcount ratio for Ethiopia further corroborates the challenges on affordability. Accordingly, when the costs of single day treatment for the common infections are benchmarked with the daily income of the population living under poverty line in Ethiopia, infections like amoebic liver abscess, meningitis and HAP cost more than this daily income of the poor even in the public sector (see Table 2). It is conceivable though that these people living under poverty line cannot spend the whole of their daily income on medications. In line with this, the impoverishing effect of essential medicine purchases whereby patients in low and middle income countries get pushed further into poverty because of unaffordability has been reported in a study elsewhere [21]. Generally, there exists a dual-faceted problem in developing countries when it comes to the utilization of antibiotics. On one hand, antibiotics can be easily purchased without prescriptions [46] and used inappropriately thereby contributing to resistance. On the other hand, patients who need antibiotics may not get them because of affordability problems. As a result, patients may purchase only the amount of antibiotics that they afford instead of what they actually need for the complete course of treatment, and this ultimately contributes to resistance [47].

Policy instruments like promoting the use of quality-assured generic drugs have long been effective strategy to enhance affordability with significant cost-savings [48]. The Ethiopian national drug policy also promises to encourage and incentivize generic prescription and generic substitution as strategies to enhance affordability and access to essential medicines. To this end, policy makers should use additional and effective policy instruments like establishing robust regulatory infrastructures, introducing tax holidays, tax exemptions, bank loans with discounted interest rates etc. to boost local pharmaceutical manufacturing capacity. Patients on their part should get organized to influence providers and policy makers. Hospitals should design internal systems like RDF that can help them better finance their drug supply thereby ensuring the availability and affordability of essential medicines.

### Conclusion

This study showed that standard treatments of commonly diagnosed infections at TASH are unaffordable; suggesting a challenge for the national drug policy promise.

## Limitations

The study did not look further into the price components and how the supply chains affected the final patient-level prices of antibiotics.

## Additional files

**Additional file 1: Table S1.** Availability of the commonly prescribed antibiotics in the private and public pharmacies around TASH, Addis Ababa (April 2015).

**Additional file 2: Table S2.** Number of day’s wages needed by the lowest paid unskilled Ethiopian government worker to purchase antibiotics for standard treatments.

## Abbreviations

CAP: Community Acquired Pneumonia
DU90%: drug utilization 90%
ETB: Ethiopian Birr
HAI: Health Alliance International
HAP: Hospital Acquired Pneumonia
IDSA: Infectious Disease Society of America
IRP: International Reference Prices
MPR: median price ratio
MSH: management science for health
NEML: National Essential Medicines Lists
RDF: revolving drug fund
SBE: sub-acute bacterial endocarditis
TASH: Tikur Anbessa Specialized Hospital
USD: United States Dollar
UTI: urinary tract infection
WHO: World Health Organization
