# Supplementary material for: Affordability of commonly prescribed antibiotics in a large tertiary teaching hospital in Ethiopia: a challenge for the national drug policy objective
Source: BMC Res Notes. 2018 Dec 27;11:925. doi: 10.1186/s13104-018-4021-2 (PMC6307120; doi:10.1186/s13104-018-4021-2)
Supplement: Supplementary file 1 — Additional file 1: Table S1. Availability of the commonly prescribed antibiotics in the private and public pharmacies around TASH, Addis Ababa (April 2015). [file 13104_2018_4021_MOESM1_ESM.docx]

Table_S1: Availability of the commonly prescribed antibiotics in the private and public pharmacies around TASH, Addis Ababa (April 2015).

| Antibiotic | Percentage of pharmacies where antibiotic was found | |
| --- | --- | --- |
|  | Private (n=9) | Public (n=5) |
| Ceftriaxone 1000mg Vial | 100% | 100% |
| Metronidazole 500mg Vial | 100% | 100% |
| Ciprofloxacin 500mg Tab | 100% | 100% |
| Vancomycin 500mg Vial | 88.9 % | 100% |
| Co-trimoxazole 400/80mg Tab | 77.8% | 100% |
| Azithromycin 500mg Tab | 88.9% | 100% |
| Ampicillin 1000mg Vial | 88.9% | 100% |
| Ceftazidime 1000mg Vial | 88.9% | 80% |
| Gentamycin 80mg/2ml Amp | 88.9% | 100% |
| Amoxycillin 500mg Cap | 100% | 100% |
| Cloxacillin 500mg Vial | 88.9% | 100% |
| Doxycycline 100mg Cap | 88.9% | 100% |
| Erythromycin 500mg Tab | 100% | 100% |
| Average | 92.3% | 98.5% |
