# Supplementary material for: Affordability of commonly prescribed antibiotics in a large tertiary teaching hospital in Ethiopia: a challenge for the national drug policy objective
Source: BMC Res Notes. 2018 Dec 27;11:925. doi: 10.1186/s13104-018-4021-2 (PMC6307120; doi:10.1186/s13104-018-4021-2)
Supplement: Supplementary file 2 — Additional file 2: Table S2. Number of day’s wages needed by the lowest paid unskilled Ethiopian government worker to purchase antibiotics for standard treatments. [file 13104_2018_4021_MOESM2_ESM.docx]

| **Infectious condition** | **Antibiotics, strength/dispensing unit** | **Treatment schedules** | **Days of wages needed to pay for the full course of antibiotic treatment** | | | | | |
| --- | --- | --- | --- | --- | --- | --- | --- | --- |
|  |  |  | **Private Pharmacies** | | | **Public Pharmacies** | | |
|  |  |  | Highest price | Median price | Lowest price | Highest price | Median price | Lowest price |
| Sub-acute bacterial endocarditis (SBE) | Ceftriaxone, 1000mg Vial  PLUS | 2g IV, every 24hrs for 45 days | 387 | 117 | 85.5 | 112.5 | 54 | 49.5 |
|  | Gentamycin, 80mg/2ml Amp | 3mg/kg every 24 hrs for 15 days |  |  |  |  |  |  |
| Amoebic liver abscess | Metronidazole, 500mg vial | 500mg IV, every 6hrs for 10 days | 44 | 36 | 24 | 60 | 32 | 24 |
| Urinary tract infection-Acute, uncomplicated in women | Ciprofloxacin, 500mg Tab | 500mg PO, every 12 hrs for 3 days | 3 | 0.6 | 0.6 | 2.4 | 0.6 | 0.2 |
| Meningitis | Ceftriaxone, 1000mg Vial  PLUS | 2g IV, every 12hrs for 14 days | 599.2 | 420 | 369.6 | 291.2 | 246.4 | 196 |
|  | Vancomycin, 500mg Vial | 1g IV, every 12hrs for 14 days |  |  |  |  |  |  |
| Sepsis | Cloxacillin, 500mg Vial | 500mg IV, every 6hrs for 10 days | 16 | 12 | 12 | 16 | 8 | 8 |
| Community Acquired Pneumonia (CAP) | Ceftriaxone, 1000mg Vial | 1g IV, every 12 hrs for 7 days | 56 | 16.8 | 12.6 | 14 | 7 | 7 |
| Hospital Acquired Pneumonia (HAP) | Ceftazidime, 1000mg Vial  PLUS | 1g IV, every 8hrs for 14 days | 1433.6 | 768.6 | 541.8 | 613 | 415.8 | 331.8 |
|  | Vancomycin | 1g IV, every 12 hrs for 14 days |  |  |  |  |  |  |
| Acute diarrheal disease | Doxycycline, 100mg Cap | 100mg PO, every 12 hrs for 3 days | 3 | 0.6 | 0.2 | 3 | 0.2 | 0.1 |
| Spontaneous bacterial peritonitis (SBP) | Ceftriaxone, 1000mg Vial | Ceftriaxone, 1000mg IV every 12 hrs for 10 days | 80 | 24 | 18 | 20 | 10 | 10 |
| Rheumatic Fever | Azithromycin, 250mg Tab | 500mg 1^st^ day and 250mg daily for 4 days | 5.4 | 3.6 | 2.7 | 4.5 | 3.9 | 1.8 |
| Toxoplasmosis | Co-trimoxazole, 480 (80/400)mg Tab | 320/1600mg P.O., every 12 hrs for 30 days, then 160/800mg P.O. every 12 hrs for 90 days  ***Followed by*** Maintenance treatment with 160/800mg P.O., every 24 hrs for a week | 61.4 | 12.3 | 6.1 | 12.3 | 12.3 | 6.1 |

Table_S2: Number of day's wages needed by the lowest paid unskilled Ethiopian government worker to purchase antibiotics for standard treatments
